# Variations in the Biological Functions of HIV-1 Clade C Envelope in a SHIV-Infected Rhesus Macaque during Disease Progression

**DOI:** 10.1371/journal.pone.0066973

**Published:** 2013-06-26

**Authors:** For Yue Tso, Levon Abrahamyan, Shiu-Lok Hu, Ruth M. Ruprecht, Charles Wood

**Affiliations:** 1 Nebraska Center for Virology and the School of Biological Sciences, University of Nebraska-Lincoln, Lincoln, Nebraska, United States of America; 2 Department of Pharmaceutics, University of Washington, Seattle, Washington, United States of America; 3 The Washington National Primate Research Center, University of Washington, Seattle, Washington, United States of America; 4 Dana-Farber Cancer Institute, Boston, Massachusetts, United States of America; 5 Harvard Medical School, Boston, Massachusetts, United States of America; University of Missouri, United States of America

## Abstract

A better understanding of how the biological functions of the HIV-1 envelope (Env) changes during disease progression may aid the design of an efficacious anti-HIV-1 vaccine. Although studies from patient had provided some insights on this issue, the differences in the study cohorts and methodology had make it difficult to reach a consensus of the variations in the HIV-1 Env functions during disease progression. To this end, an animal model that can be infected under controlled environment and reflect the disease course of HIV-1 infection in human will be beneficial. Such an animal model was previously demonstrated by the infection of macaque with SHIV, expressing HIV-1 clade C Env V1-V5 region. By using this model, we examined the changes in biological functions of Env in the infected animal over the entire disease course. Our data showed an increase in the neutralization resistance phenotype over time and coincided with the decrease in the net charges of the V1-V5 region. Infection of PBMC with provirus expressing various Env clones, isolated from the infected animal over time, showed a surprisingly better replicative fitness for viruses expressing the Env from early time point. Biotinylation and ELISA data also indicated a decrease of cell-surface-associated Env and virion-associated gp120 content with disease progression. This decrease did not affect the CD4-binding capability of Env, but were positively correlated with the decrease of Env fusion ability. Interestingly, some of these changes in biological functions reverted to the pre-AIDS level during advance AIDS. These data suggested a dynamic relationship between the Env V1-V5 region with the host immune pressure. The observed changes of biological functions in this setting might reflect and predict those occurring during natural disease progression in human.

## Introduction

Despite years of efforts, there is no efficacious vaccine against HIV-1. This impasse is partly due to the highly variable nature of the HIV-1 envelope gene, which enables viral escape from immune surveillance [1]. Most of the envelope diversity is within the V1-V5 region, which encompasses multiple crucial functions, such as binding to receptor and co-receptors, and can influence envelope fusion to the cell membrane [2], [3], . Being the major surface component of HIV-1 envelope, V1-V5 is also constantly targeted by host humoral responses [6], [7]. Given these important roles, the V1-V5 region is frequently targeted for diverse anti-HIV strategies. Understanding the changes of HIV-1 envelopes *in vivo* may allow prediction of how the envelope will evolve in the context of host immune suppression, which will be of substantial importance for future vaccine and drug designs. The first step towards achieving this goal is to examine how the biological functions of HIV-1 V1-V5 region evolve during disease progression. Unfortunately, there is no consensus as to what these changes might be, due to the difficulties in following individuals from initial HIV-1 infection throughout disease progression and differences in methodology.

To address this question, we have previously shown a strikingly similar molecular evolution of the HIV-1 clade C (HIV-C) envelope during infection in a Zambian infant (1157i) and an infant rhesus macaque (RPn-8) infected by SHIV-1157i, a SHIV expressing the recently transmitted HIV-C envelope of 1157i [8]. This finding validated our macaque model for studying envelope evolution during disease progression. Importantly, our macaque model allows studying the longitudinal changes of the biological functions of the V1-V5 region from an infectious molecular clone inoculum over the entire disease course. Moreover, significant mutations were observed within the HIV-C envelope sequences from different disease stages in the infected macaque RPn-8 [8]. However, whether these envelope mutations translate into different biological functions remains unanswered. Here, we addressed this question by characterizing the biological properties of the V1-V5 region cloned from RPn-8 during disease progression. Our hypothesis is that envelopes from various disease stages have different biological characteristics, which might confer fitness advantages for the virus.

This study will be of substantial importance since it is the first reported clade C SHIV (SHIV-C) model that documented progression to AIDS. Moreover, the recently transmitted Zambian HIV-C primary isolate we utilized is CCR5 tropic and represents the dominant clade of HIV-1 infection worldwide [9], [10], [11], [12]. The observed changes in biological functions in this setting should reflect and predict those occurring during natural disease progression.

## Results

### Charged Residues of the HIV-C V1-V5 Region

Charged residues play an important role in protein-protein interactions by affecting the physiological interactions between molecules, and might influence HIV-1 envelope-antibody interaction, receptor binding as well as disease progression [13], [14], [15], [16]. Therefore, it was interesting to determine whether the charges of V1-V5 region varied with disease progression in monkey RPn-8.

**Figure 1:**
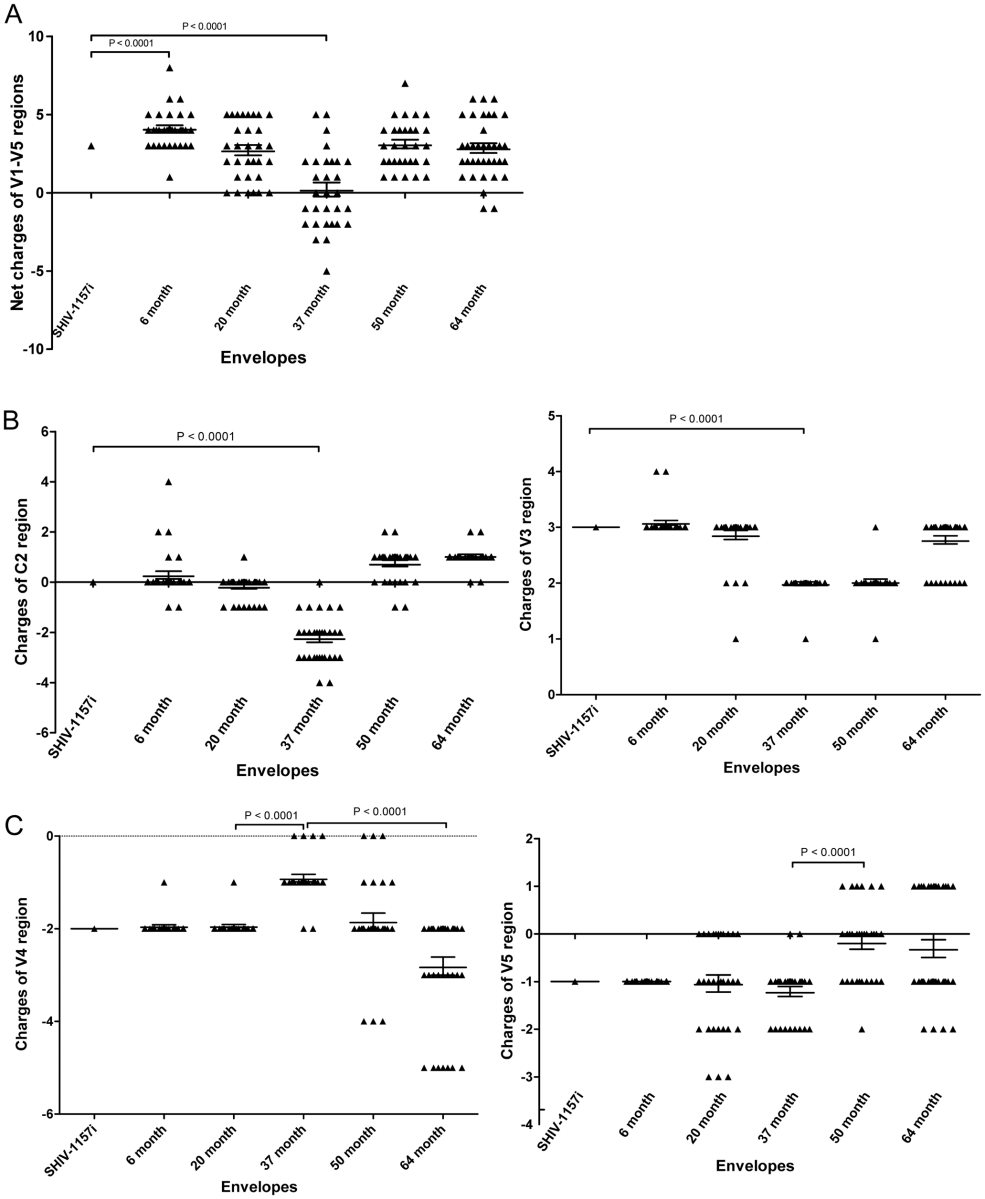
Distribution of charged residues within the V1-V5 envelope region. Each symbol represents one V1-V5 sequence. SHIV-1157i is the inoculum envelope, followed by envelopes from 6, 20, 37, 50 and 64 months pi. (A) Net charge of the V1-V5 region. (B) Charge distribution within the C2 and V3 region. (C) Charge distribution within V4 and V5 region.

As shown previously, RPn-8 maintained a high viral load (>1×10^4^ copies/ml) throughout the disease course [8] and the CD4^+^ T-cell counts gradually declined to <200 cells/ul from ∼28 mpi onwards. The animal was euthanized at 64 mpi due to severe opportunistic infections [8]. Using ∼160 sequences collected previously across the entire disease course [8], we analyzed the charged residue distribution within the V1-V5 region. Our analysis showed a significant increase in the net charge of V1-V5 region during early infection (6 mpi) compared to the inoculum (Fig. 1A). However, the net charge decreased substantially by 37 mpi when the CD4^+^ T-cell counts fell to <200 cells/ul. Surprisingly, as RPn-8 had advanced AIDS, the net charge of the V1-V5 region recovered to pre-AIDS levels.

This pattern of change was mainly caused by the V1-V3 region. For example, C2 and V3 charges reached the lowest levels by 37 mpi but rebounded later (Fig. 1B). Similar pattern was reported within the V1V2 of RPn-8 [17]. Other regions, such as V5, contributed towards the recovery of envelope charges during advanced AIDS, which was often accompanied by decreases in regions such as V4 (Fig. 1C). The V1-V5 charges of envelope clones for subsequent functional analysis are representative to the envelope population in each time point (Fig. S1).

### Neutralization Characteristics

To determine the neutralization characteristics of the V1-V5 region, pseudotype viruses expressing selected V1-V5 Env clones were tested against naïve, contemporaneous and non-contemporaneous plasma from RPn-8. A cell-to-cell fusion assay was first used to screen out completely non-functional envelope clones. All functional envelope clones, regardless of their level of cell-to-cell fusion, were then selected based on their position in the phylogenetic tree to maximize the representation of various branches. About five functional envelope clones per time point were selected for further analysis [8].

**Figure 2:**
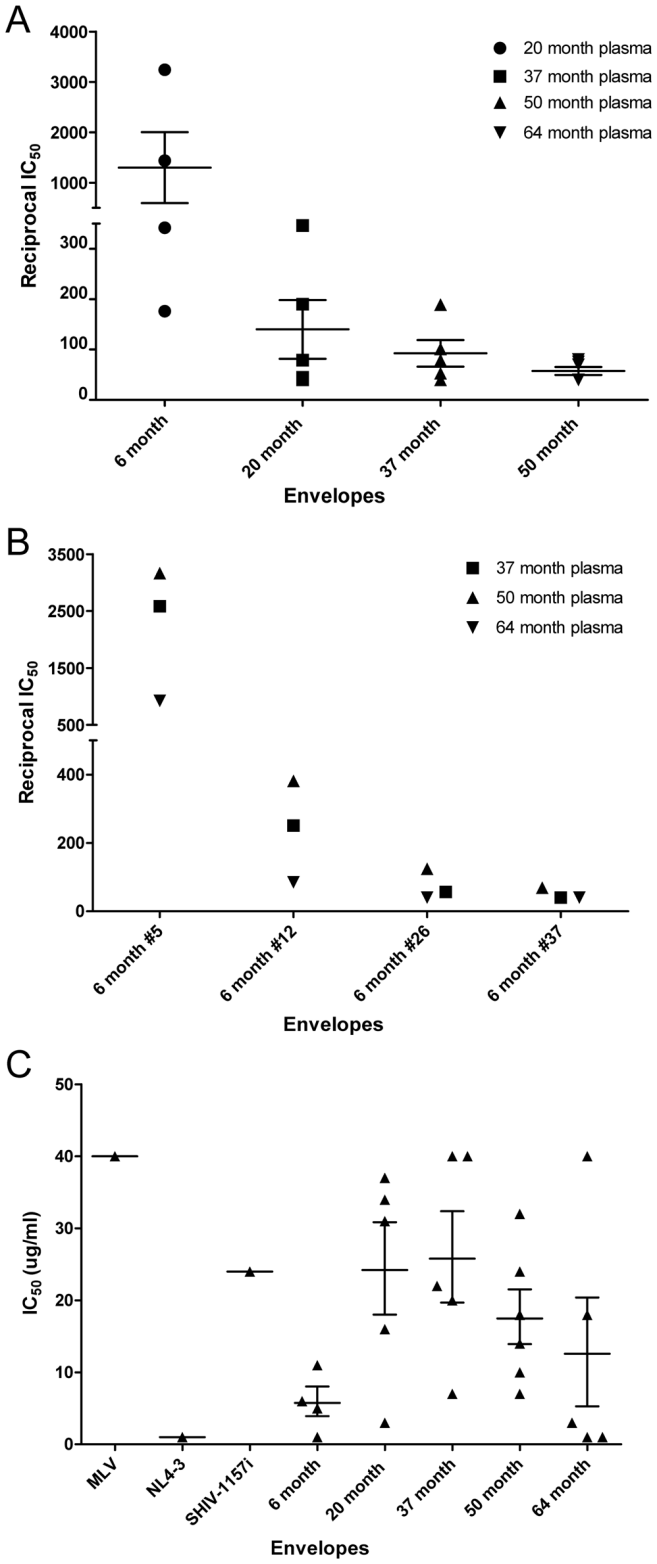
Envelope neutralization characteristics. (A) Pseudotyped viruses with envelopes from 6 months pi were tested against non-contemporaneous plasma from 20 months pi, 20 months pi were tested against non-contemporaneous plasma from 37 months pi, 37 months pi envelope clones against 50 months pi plasma and 50 months pi envelope clones against 64 months pi plasma. Each symbol represents a pseudotyped virus encoding an individual envelope clone from that particular time point. (B) Pseudotyped viruses with envelopes from 6 month pi were tested against plasma of 37, 50 and 64 months pi. (C) Pseudotyped viruses with envelopes from various time points were tested against pooled mAbs. Envelopes from the infectious molecular clone inoculum (SHIV-1157i), murine leukemia virus (MLV) and NL4-3 served as controls. Each symbol represents a pseudotyped virus encoding an individual envelope clone from that particular time point.

We found that neither the naïve nor the contemporaneous plasma could neutralize any of the envelopes tested, including the inoculum SHIV-1157i (data not shown). However, non-contemporaneous plasma was able to neutralize envelopes from earlier time points, but this capability tended to decrease with disease progression (Fig. 2A). This was further confirmed by the examination of 6 mpi envelopes against plasma samples from all later time points, where the 64 mpi plasma had the weakest neutralizing ability (Fig. 2B).

Since plasma from RPn-8 might contain different neutralizing antibodies, depending on the time points, a reference panel that consisted of pooled mAbs was used to examine the relative neutralization sensitivity among the envelopes. Our data show that envelopes from 6 mpi were mostly sensitive to mAbs (Fig. 2C). However, the majority of the envelopes displayed increased mAbs resistance by 37 mpi. Surprisingly, mAbs-sensitive envelopes re-emerged at 64 mpi.

### Replication Kinetics

To determine if envelopes derived from different disease stages could affect the viral replicative fitness and its relationship with neutralization sensitivity, proviruses expressing various envelope clones were generated and their replication kinetics were examined in PBMC. In addition to the inoculum SHIV-1157i envelope, nine envelope clones were selected from the 6 (early infection), 37 (early AIDS) and 64 (advanced AIDS) mpi. Envelope selection was based on the mAbs neutralization results to maximize representation of different neutralization characteristics. As shown in Table 1 (Fig. S2), viruses with 6 month envelopes (clones #26 and #37) displayed distinctively faster *ex-vivo* replication (P = 0.0008) than those with 64 mpi envelopes, averaging ∼7 days to reach half-maximal viral replication using PBMC from two human donors. In contrast, viruses bearing envelopes from later time points showed slightly slower *ex-vivo* replication compared to the inoculum over time.

**Table 1:** *Ex-vivo* replication of infectious viruses, expressing varies V1-V5 region, in PBMC.

	Half-time of maximal viral replication (days)	
Envelopes	Donor 1	Donor 2
**SHIV-1157i**	9.739	8.951
**6 month #5**	10.032	10.798
* **6 month #26**	7.099	7.083
* **6 month #37**	6.510	6.315
**37 month #1**	10.555	10.813
**37 month #11**	8.998	9.797
**37 month #S1**	11.207	12.258
**64 month #13**	11.922	15.105
**64 month #30**	9.880	11.130
**64 month #46**	10.414	12.496

* Denote statistical significances (P = 0.0008) in comparison with the 64 month envelopes.

### Quantity of Envelope on Cell Surfaces and gp120 on Virions

**Figure 3:**
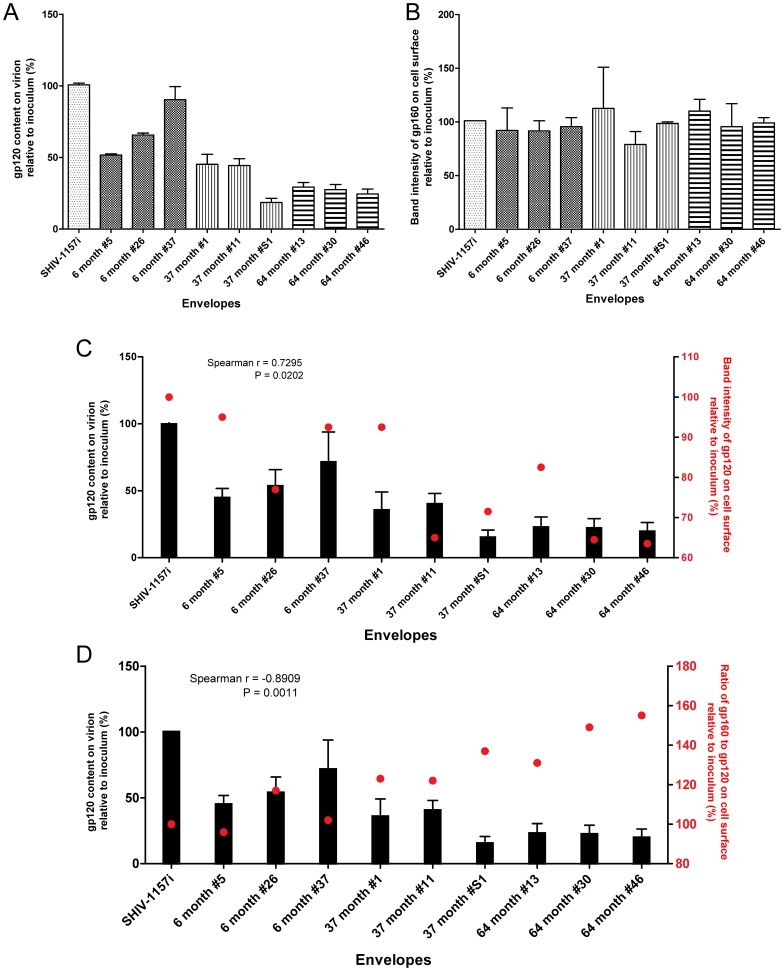
Quantity of envelope on cell surfaces and gp120 on virions. (A) Quantities of gp120 on virions expressing selected V1-V5 region from each time point. (B) Relative levels of 293T cell surface-associated gp160. (C) Correlation between the levels of virion-associated gp120 (black bars) and 293T cell surface-associated gp120 (red dots). (D) Correlation between the levels of virion-associated gp120 (black bars) and the ratio of 293T cell surface-associated gp160–120 (red dots).

To further examine the possible reasons for faster *ex-vivo* replication of the viruses with early envelopes, we first evaluated the incorporation of envelopes into virions. By measuring the gp120 content on virions using ELISA, we showed that the quantity of surface glycoprotein on virions tended to decline with disease progression. Viruses expressing the 64 mpi envelopes only contained an average ∼25% (P<0.0001) of gp120 content compared with those expressing the inoculum SHIV-1157i envelope (Fig. 3A).

This decreased level of virion-associated gp120 could be caused by differences in the expression or cleavage of envelope. To eliminate these possibilities, 293T cell surfaces were labeled with biotin, immunoprecipitated and the quantity of cell surface-associated gp160 and 120 was measured by Western blot (Fig. S3). Our data indicated no significant differences in gp160 expression levels between all envelopes (Fig. 3B); thereby excluding differences in gp160 expression on cell surfaces as a plausible cause. However, we did observe a decrease in cell surface-associated gp120 levels, which positively correlated with the decline of gp120 content on virions over time (Spearman r = 0.7295, P = 0.0202) (Fig. 3C). Therefore, the decreased recruitment of surface glycoprotein onto virions may have been a consequence of having less cell surface-associated gp120, which subsequently caused an increase of the cell surface gp160 to 120 ratio over time (Fig. 3D). This was further supported by the negative correlation (Spearman r = -0.8909, P = 0.0011) between the cell surface gp160 to 120 ratio and gp120 quantity on virions (Fig. 3D). Together, our data strongly suggest a deficiency in cleavage or trafficking of cleaved envelope to cell surfaces with disease progression.

### Envelope Binding to CD4

**Figure 4:**
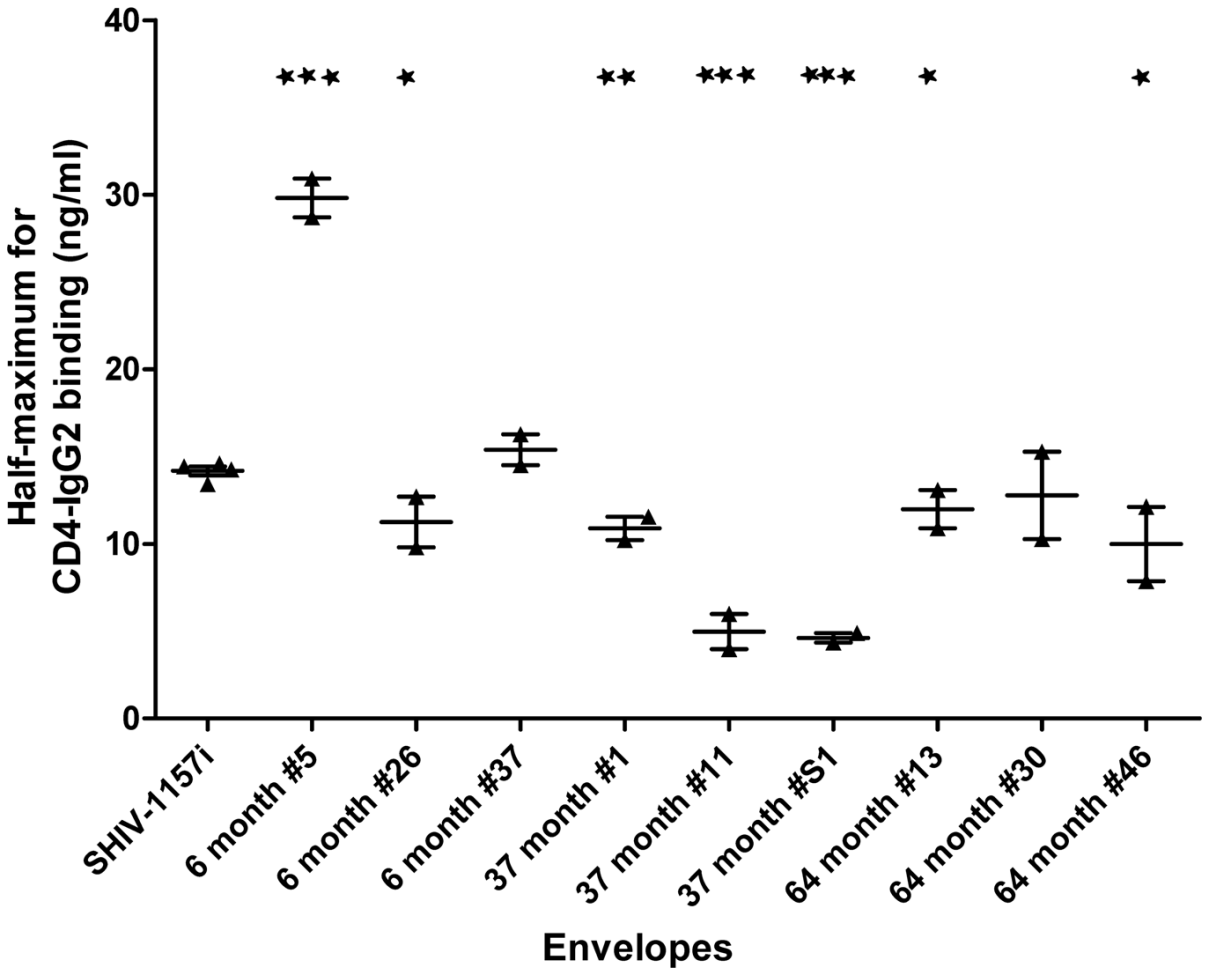
Envelope binding to CD4 and virus fusion with target cells. Purified viruses, expressing selected V1-V5 regions from each time point, were tested with *in-vitro* CD4 binding assay to determine their half maximum binding to CD4-IgG2. Each symbol for individual envelope clones represents an independent experiment. “*” represent statistical significant compare to the inoculum, SHIV-1157i. The value of 6 month #5 may not reflect the actual data since it falls beyond the maximum range of our assay.

Another possible factor that can influence *ex-vivo* replication is divergence in the envelope-CD4 binding capacity. To this end, our *in-vitro* CD4 binding assay showed that the CD4 binding proficiency of envelope improved with disease progression. At 37 mpi, the envelopes had significantly enhanced binding to CD4 compared with the inoculum (Fig. 4). Surprisingly, this enhanced CD4 binding capability was not maintained and reverted to pre-AIDS levels during advanced AIDS.

### Envelope Fusion to Target Cell

**Figure 5:**
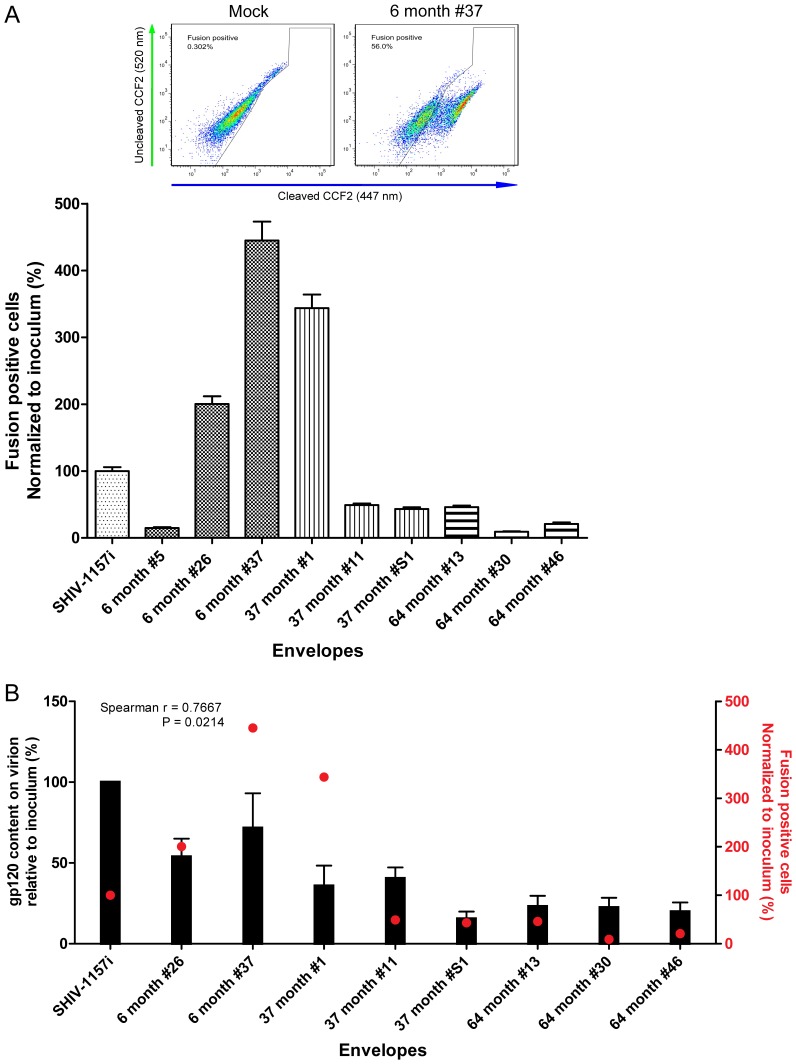
Envelope fusion with target cells was measured using FRET-based virus-to-cell fusion assay. (A) An example for the detection of fluoresce emission shift from green (fusion negative) to blue (fusion positive) by flow cytometry was shown above the graph. The graph shows percentage of fusion-positive cells for individual envelope clones from each time point relative to the inoculum, SHIV-1157i. All differences were statistically significant compared to the inoculum. (B) Correlation between the levels of virion-associated gp120 (black bars) and fusion-positive cells (red dots). The 6 month #5 is excluded from this correlation analysis since it is a CD4 binding site mutant.

Fusion of viral envelope to target cell is a critical rate-limiting step that could affect *ex-vivo* replication. A FRET-based virus-to-cell fusion assay was used to examine the fusion efficiency of envelopes from various time points. Our result showed that viruses expressing the 6 mpi envelopes, except #5, had significantly enhanced fusion abilities compared with the inoculum (P = <0.0001) (Fig. 5A). Envelopes isolated from 37 mpi displayed a diverse fusion capability, with 37 month #1 having fusion ability close to the 6 mpi and the rest having lower fusion ability compared to the inoculum (Fig. 5A). Additionally, this weak fusion phenotype dominated among envelopes isolates from advanced AIDS (Fig. 5A). This suggests that envelope fusion ability increased during early infection, but gradually declined with disease progression.

## Discussion

This study showed that the V1-V5 regions of HIV-C envelope underwent significant changes in their biological characteristics throughout disease progression. Envelopes from early infection were more positively charged, neutralization sensitive and had faster *ex-vivo* replication and enhanced fusion capability. During early AIDS, the envelopes became more negatively charged with diminished functional capabilities ranging from *ex-vivo* replication to incorporation into virions. However, these envelopes did have significantly improved CD4 binding ability and were more neutralization resistant. Interestingly, the envelopes showed multiple reversions in their biological properties during advanced AIDS. For example, their charges, neutralization sensitivity and CD4 binding efficiency had all reverted to pre-AIDS levels. However, other functions remained relatively poor.

Our results are surprising given the current belief that viral fitness increases over time due to envelope evolution [18], [19]. It is possible that the previous studies were unable to capture the early infection in adult HIV-1 patients and used PBMC co-cultured viral quasi-species for biological analysis. Our study represents envelope isolates throughout the disease spectrum, focusing on V1-V5 region changes. At the early infection of 6 mpi, monkey infant RPn-8 had an unambiguously higher CD4^+^ T-cell counts of >2000 cells/ul, which was age-appropriate, and high viral loads compared to reports of other investigators, reflecting acute infection [8]. Moreover, having an isogenic backbone allowed us to focus on the V1-V5 region without the influences from other viral genes. Our use of PBMC for *ex-vivo* replication also better represents the *in-vivo* scenario than the cell lines used in previous study [2]. Alternatively, the different maturation state of the immune system between adults and infants during the initial infection might affect the evolutionary pressure placed on the virus.

Our study suggests that during early infection, the host immune system is intact and exerts selective pressures on the viruses, as the relatively early plasma can easily neutralized the early envelopes. To counter this host constraint, the envelope evolved compensatory mutations that resulted in more efficient fusion and faster replication kinetics, increasing the likelihood for the virus to infect new cells before being neutralized by the immune system. This possibility that early envelopes might have better fitness was mathematically predicted previously and observed recently in HIV-1 patients [20], [21]. Although the exact mechanism of this outcome is still controversial, the “Red Queen” hypothesis was proposed as a possible explanation, where beneficiary mutations evolved in responses to immune selection resulting in increased fitness of the early envelopes [21], [22], [23].

During early AIDS, some immune pressure persisted as evident by the presence of neutralization-resistant envelopes and was also reported by others [13], [24]. Importantly, the appearance of neutralization-resistant phenotypes coincided with a significant drop in the net charges of V1-V5 region. This strongly suggests that variations in net charges could be an escape mechanism employed by the envelope to evade humoral responses beside its glycan shield [7]. The high CD4 binding ability of the envelopes at this stage could be another compensatory mutation for more efficient infection in an environment with limited availability of CD4^+^ T cells.

Another unexpected finding from our study is that viral fitness linked to the V1-V5 region, as defined by the *ex-vivo* replication of isogenic viruses differing only in their V1-V5 regions, did not improve despite reversion of several envelope properties during advanced AIDS. Importantly, the gp120 content on cell surface and on virion gradually decreased as disease progressed. Additionally, with the exception of the CD4 binding site mutant 6 month #5, decreased levels of gp120 on virion correlated with the decline in fusion ability from early infection to advanced AIDS (Spearman r = 0.7667, P = 0.0214) (Fig. 5B). These changes could be evasive responses toward immune surveillance and ultimately affected the neutralization sensitivity as suggested earlier [25]. The severe depletion of CD4^+^ T cells and absence of immune selection might facilitate the accumulation of deleterious mutations, possibly yielding envelopes with lower fitness reminiscent to the effect of “Muller’s ratchet” hypothesis [26], [27], [28], [29]. The lack of immune surveillance during the advanced AIDS might allow accumulation of viruses with lower fitness envelopes, resulting in the increase of plasma viral load.

Our study is based on retrospectively collected specimens from an animal that developed AIDS and no other specimens or animals were available for further analysis, the changes we observed could be only confined to this animal. However, the lessons we learned from this study cannot be underestimated, as this is the first study of a SHIV-C-infected macaque that progressed to AIDS. To our knowledge, this is also the first study that demonstrated a decrease of gp120 content on virion with disease progression. Although gp120 shedding might reduce the gp120 content on virion, it was unlikely in our case as suggested by other investigators [30]. By focusing on the V1-V5 region, we cannot eliminate the role of other viral genes or envelope regions, such as the signal peptide and gp41, in the overall viral fitness [31]. However, our data strongly suggest that V1-V5 region plays a major role in disease progression [32], [33]. Also, the presence of mixed population at any time points due to reactivation of latent viral reservoirs cannot be excluded.

Our findings underscore the dynamic interaction between host immune selection and HIV-1 envelope evolution. Our emphasis on the V1-V5 region had shed light on the evolution of its unique properties during disease progression. Moreover, variations in the envelope CD4 binding and fusion ability might suggest some corresponding structural changes with disease progression, which could have substantial implications for the development of vaccines and small molecule inhibitors.

## Materials and Methods

### Animal and Animal Care

An infant Indian rhesus macaque (RPn-8) was intravenously inoculated with SHIV-1157i, a SHIV-C expressing the envelope of a recently transmitted Zambian pediatric HIV-C. The infected animal was monitored from inoculation until euthanasia due to AIDS (∼5 years). Details of animal care and procedures were described [8], [34]. The animal was housed and cared for according to the National Institutes of Health Guidelines on the Care and Use of Laboratory Animals at the Yerkes National Primate Research Center (YNPRC), which is fully accredited by the Association for Assessment and Accreditation of Laboratory Animal Care International. The animal is kept in two level Allentown stainless steel cage racks commensurate with the size and needs of the animal. The animal cage pans are cleaned daily with weekly cage changes. The animal quarter is maintained with controlled air humidity and quality, as well as a 12 hour on/off schedule for access to light. Commercial monkey chow is fed to the animal daily with supplemented fresh fruit or vegetable and drinking water is available at all times. Environmental enrichment is provided daily in the form of devices that provide foraging opportunities and toys such as kongs. The animal is monitored at least twice a day with medical care provided by Yerkes staff veterinarians. All sample collections were performed under ketamine/Telazol anesthesia to alleviate any pain or stress and recovery of the animal was closely monitored. Euthanasia of the animal is performed in accordance with recommendations of the American Veterinary Medical Association using an overdose of pentobarbital. Humane endpoints have been defined by the Emory IACUC and include perimeters such as weight loss exceeding 25% and untreatable disease that cannot be alleviated therapeutically. All animal procedures were approved by the Animal Care and Use Committees of the YNPRC and the Dana-Farber Cancer Institute.

### Construction and Preparation of Viruses

The SHIV-1157i inoculum used for the infection of macaque was prepared as described [34], [35]. Genomic DNA was extracted from PBMC samples of RPn-8 collected at 6, 20, 37, 50 and 64 months post-infection (mpi). The V1-V5 region was amplified from genomic DNA and cloned into the envelope expression (pSRH NLA/S/Av) and provirus (pNL4-3 A/S/Av) plasmids as described [8], [36], [37]. Thus, both types of plasmids bear a chimeric HIV-1 NL4-3 envelope with its V1-V5 region derived from RPn-8. Importantly, this mirrors the chimeric nature of the inoculum, SHIV-1157i, which consists of the HIV-1 HXBc2 and clade C envelope [35]. Infectious virus was generated by transfection of 293T cells with the proviral plasmids, supernatant was collected and filtered at 48 hrs post-transfection (pt). For purified virus, the filtered supernatant was concentrated by ultracentrifuge and the resulting virus pellet was re-suspended in their respective assay medium.

### Envelope Charge Analysis

V1-V5 region sequences were obtained from the previous study [8]. Charged residues of the envelope were computed using AminoTrack™ [38]. The value of +1 was assigned to positively charged arginine and lysine. The value of -1 was assigned to negatively charged aspartic acid and glutamic acid. GraphPad Prism 5 (GraphPad Software) was used for statistical analysis.

### Neutralization Assay

Pseudotyped viruses were generated by co-transfection of COS-1 cells with the envelope expression and backbone pNL4-3-deltaE-GFP plasmids, supernatants were collected and filtered at 48 hrs pt. Pseudotyped virus encoding NL4-3 and SV-A-MLV envelopes served as controls. Next, 1×10^4^ TZM-bl cells/well were seeded into 96-well plates 24 hrs before the assay. Heat-inactivated plasma samples of RPn-8 were 3-fold serially diluted starting from 1∶20. Pooled monoclonal antibodies (mAbs) were prepared by mixing equal concentrations of the broadly neutralizing anti-HIV-1 mAbs 2F5, 4E10, 2G12 and b12, followed by 3-fold serially dilutions starting from 80 ug/ml (total concentration of all mAbs in the mixture). An inoculum of 300 50% tissue culture infectious doses (TCID_50_)/ml of pseudotyped viruses was supplemented with 80 ug/ml DEAE-dextran and mixed with an equal volume of either the serially diluted heat-inactivated plasma or pooled mAbs. The mixture was incubated for 1 hr at 37°C before addition to TZM-bl cells in triplicates. After 48 hrs incubation at 37°C, the cells were washed, lysed with Luciferase Assay System reagents (Promega) and its luciferase activity measured by luminometer. The 50% inhibitory concentration (IC_50_) in ug/ml and reciprocal IC_50_ were calculated using GraphPad Prism 5.

### Viral Replication Kinetics

Infectious viruses bearing the V1-V5 region of RPn-8 were used to infect PBMC from HIV-1-seronegative donors. PHA-stimulated PBMC (3.5×10^5^ cells) were infected at a multiplicity of infection (MOI) of 0.05 overnight at 37°C in duplicates. The next day, the infected cells were washed and resuspended in RPMI growth medium containing human interleukin-2 (hIL-2) (Roche Applied Science). Cell-free supernatants were collected every 3 days. The level of reverse transcriptase (RT) activity in 10 µl of collected supernatants was determined in triplicates by the standard RT activity assay and expressed in counts-per-minute (CPM) [39]. The experiment was repeated using a different HIV-1-seronegative donor PBMC.

### Quantification of Virus-associated gp120

The amount of virus-associated HIV-1 surface glycoprotein was measured using ELISA as described [40]. Briefly, 96-well plates were coated overnight with anti-gp120 antibody D7324 (Aalto Bio Reagents Ltd), followed by washing with Tris-buffered saline (TBS) and blocked with nonfat-milk solution. Purified virus (100 ng/ml p24 equivalents) was lysed and added to the plates in triplicates for 5 hrs incubation at 37°C. Unbound proteins were removed by extensive washes. For detecting captured gp120, mAb T43 (NIH) was added at room temperature. Importantly, the mAb T43 epitope lies within the envelope C1 region, which is identical among all our samples. The plates were washed and captured gp120 was detected by addition of goat anti-mouse horseradish peroxidase (Jackson ImmunoResearch Laboratories) with 1-Step Ultra-TMB-ELISA substrate (Pierce). Gp120 quantity was measured by microplate reader and calculated by comparison with a standard curve generated using serial dilutions of purified HIV-C 1157ip gp120 with a known concentration.

### Quantification of Cell Surface-associated Envelope

Labeling of cell-surface proteins with biotin was described [41]. Briefly, 293T cells transfected with proviral plasmids were harvested at 48 hrs pt, washed and incubated with 1 mg/ml of EZ-Link Sulfo-NHS-LC-biotin (Thermo Scientific) for 30 min on ice, followed by extensive washes and addition of lysis buffer containing proteinase inhibitor cocktail. Cellular debris was removed and the total protein concentration in the cleared lysates was measured using a Pierce BCA protein assay kit (Pierce). Equal amounts of total protein were used for HIV-1 envelope immunoprecipitation with HIV-IG (NIH) overnight at 4°C. The immune complexes were pulled down with Protein A/G Ultra-Link Resin (Thermo Scientific), washed and boiled in 2X SDS-PAGE sample buffers. Resin-free supernatant containing the immunoprecipitated proteins was resolved by Western blot. Biotinylated proteins were detected using streptavidin-conjugated-680 secondary antibody (Li-Cor Biosciences) and visualized by Odyssey infrared imager (Li-Cor Biosciences). Respective band intensity of the cell surface-associated HIV-1 envelope was analyzed using Odyssey application software version 3.0.

### In-vitro CD4 Binding

Envelope CD4 binding capability was determined by ELISA. Briefly, purified virus (10 ng/ml gp120 equivalents) was lysed and captured with D7324-coated plates in triplicates. Serial dilutions of CD4-IgG2 with known concentrations were added and incubated overnight at room temperature. Unbound CD4-IgG2 was removed by extensive washes. CD4-IgG2 bound to gp120 was detected by addition of goat-anti-human horseradish peroxidase (Santa Cruz Biotechnology) with 1-Step Ultra-TMB-ELISA substrate (Pierce) and measured by microplate reader. Half-maximal binding concentration (ng/ml) of CD4-IgG2 was calculated for each sample.

### FRET-based Virus-to-cell Fusion

The fluorescent resonance energy transfer (FRET)-based virus-to-cell fusion assay was described [42], [43]. Briefly, infectious viruses bearing the V1-V5 region of RPn-8 and the β-lactamase HIV-1 Vpr fusion protein (BlaM-Vpr) were generated by co-transfection of 293T cells with the provirus, pAdVantage (Promega) and pMM310 plasmids (NIH). Supernatants were collected at 48 hrs pt and purified as described earlier. The p24 concentration of purified viruses were measured with HIV-1 p24 ELISA (Perkin-Elmer). Then, 2.5×10^5^ SupT1/CCR5 cells [44] were incubated with 50 ng of p24 of the purified viruses for 2 hrs at 37°C in 96-well plates in triplicates. The infected cells were washed and resuspended in the CCF2-AM loading solution from GeneBLAzer Detection Kits (Invitrogen) for 1 hr at room temperature in the dark. The CCF2-AM-loaded cells were washed, resuspended in 10% FBS medium with probenecid and incubated overnight at room temperature in the dark. The next day, CCF2-AM-loaded cells were washed and fixed for flow cytometry. Once the BlaM-Vpr viruses fused with the target cell, β-lactamase will cleave the CCF2-AM dye and alter its fluorescence emission spectrum from 520 nm (green) to 447 nm (blue). This change in fluorescence emission was detected by Cytek DxP10 (Cytek Development) and analyzed using FlowJo software v7.9 (TreeStar). GraphPad Prism 5 was used for statistical analysis.

## Supporting Information

Figure S1 **The V1-V5 charges of envelope clones selected for various functional analysis.** Triangle (▴) symbol represent envelope clones used for neutralization assay. Envelope clones used for other functional analysis are shown in red.
(PDF)

Figure S2 **Ex-vivo replication of infectious viruses, expressing the V1-V5 region from the inoculum clone, SHIV-1157i, and varies time points.** (A) Replication kinetic in PBMC from donor JW. (B) Replication kinetic in PBMC from donor JC.
(PDF)

Figure S3 **Example of a western blot for the biotinylated and immunoprecipitated cell surface-associated HIV-1 envelope after transfection of 293T cells with the proviral constructs encoding the V1-V5 regions from the inoculum SHIV-1157i and other time points.** The data is an example of the several experiments conducted.
(PDF)
