# Supplementary figures and images for: Variations in the Biological Functions of HIV-1 Clade C Envelope in a SHIV-Infected Rhesus Macaque during Disease Progression
Source: PLoS One. 2013 Jun 26;8(6):e66973. doi: 10.1371/journal.pone.0066973 (PMC3694120; doi:10.1371/journal.pone.0066973)

Fig S1

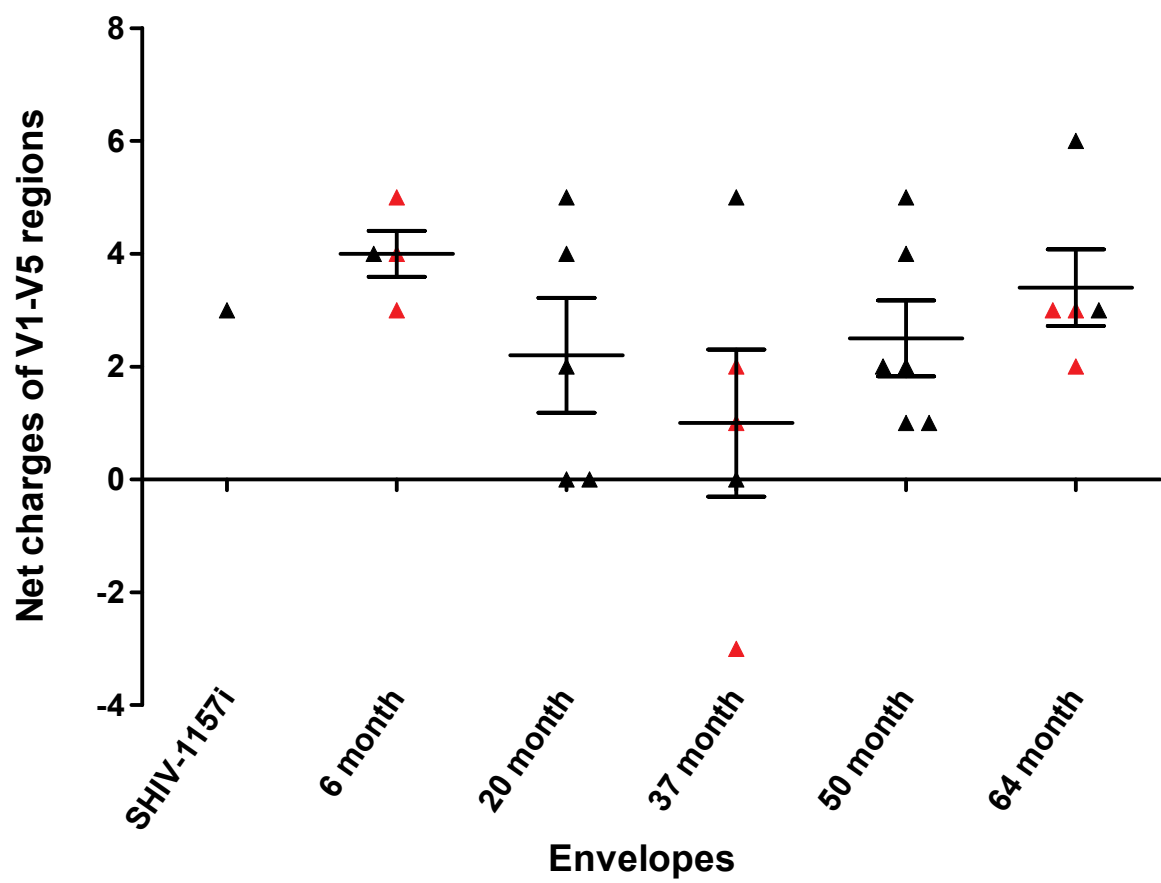

Supplement: Figure S1 — The V1-V5 charges of envelope clones selected for various functional analysis. Triangle (▴) symbol represent envelope clones used for neutralization assay. Envelope clones used for other functional analysis are shown in red. (PDF) [file pone.0066973.s001.pdf]

Fig S2

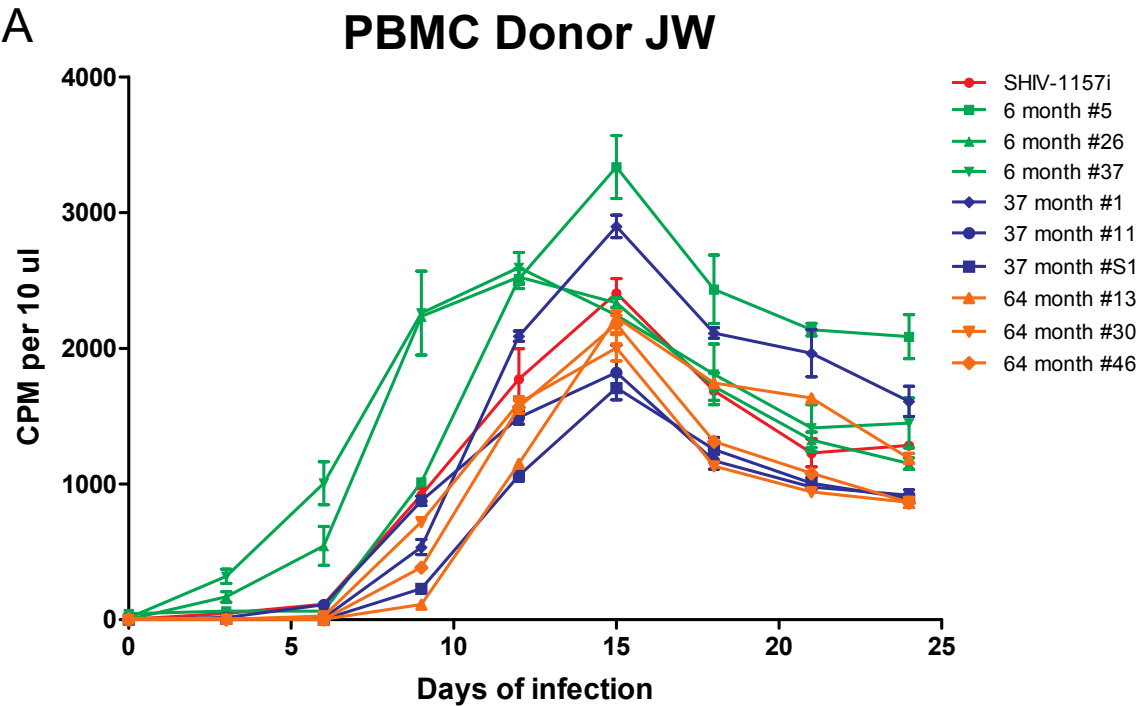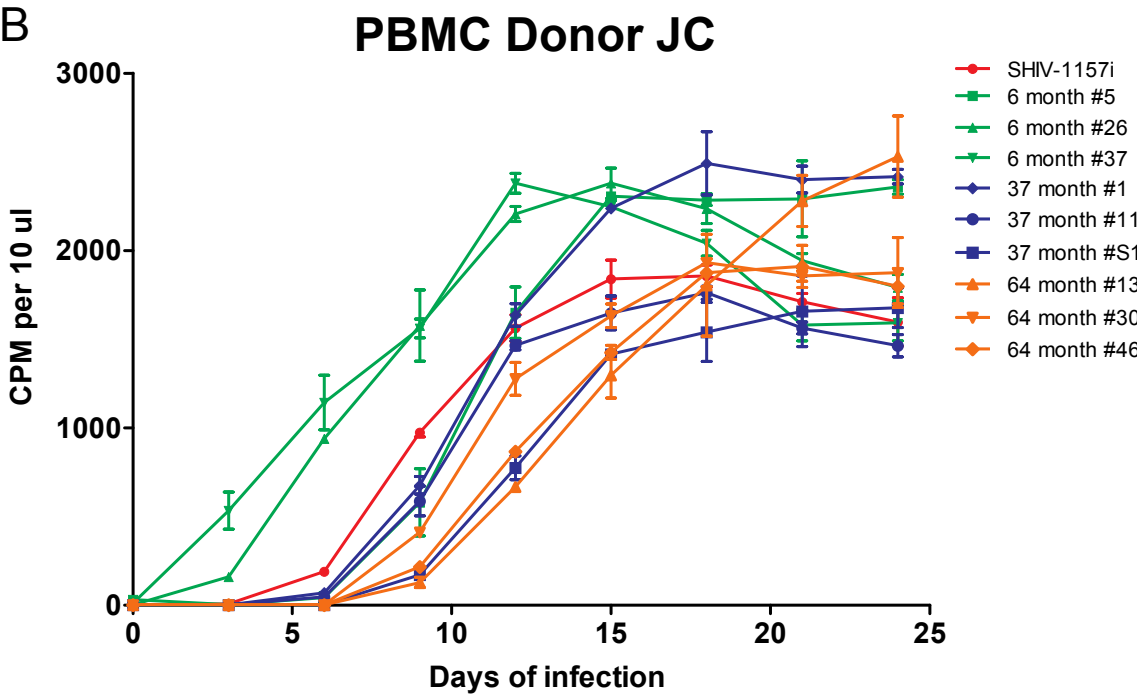

Supplement: Figure S2 — Ex-vivo replication of infectious viruses, expressing the V1-V5 region from the inoculum clone, SHIV-1157i, and varies time points. (A) Replication kinetic in PBMC from donor JW. (B) Replication kinetic in PBMC from donor JC. (PDF) [file pone.0066973.s002.pdf]

Fig S3

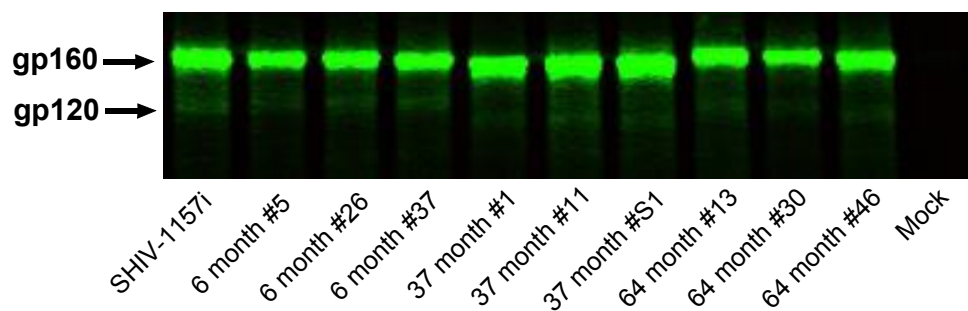

Supplement: Figure S3 — Example of a western blot for the biotinylated and immunoprecipitated cell surface-associated HIV-1 envelope after transfection of 293T cells with the proviral constructs encoding the V1-V5 regions from the inoculum SHIV-1157i and other time points. The data is an example of the several experiments conducted. (PDF) [file pone.0066973.s003.pdf]
